# Integrative analysis of Hub1 overexpression: driving transcriptional reprogramming and alternative splicing in *Saccharomyces cerevisiae*

**DOI:** 10.1186/s12864-025-12006-w

**Published:** 2025-10-06

**Authors:** N. M Asif Billah, Umama Khan, Kazi Mohammed Didarul Islam, S. M. Abdul-Awal, Md. Morsaline Billah

**Affiliations:** 1 Biotechnology and Genetic Engineering Discipline, Life Science School, Khulna University, Khulna, 9208 Bangladesh; 2 Department of Genetic Engineering and Biotechnology, Jashore University of Science and Technology, Jashore, 7408 Bangladesh

**Keywords:** Hub1, *Saccharomyces cerevisiae*, RNA-seq, Alternative splicing, *DYN2*, Transcriptomics

## Abstract

**Background:**

Hub1, a conserved ubiquitin-like protein, is essential for pre-mRNA splicing and transcriptional regulation in *Saccharomyces cerevisiae*. Despite its known functions, the genome-wide effects of Hub1 overexpression remain largely uncharacterized. This study investigates the transcriptomic and splicing landscape changes triggered by Hub1 overexpression using an integrative bioinformatic approach.

**Results:**

We analyzed RNA-seq data from the GSE84215 dataset, employing differential expression, alternative splicing, functional enrichment, and network-based methods. DESeq2 identified 3,915 differentially expressed genes (DEGs; 1,964 upregulated, 1,951 downregulated, padj < 0.05), demonstrating extensive transcriptional reprogramming. Principal component analysis revealed that Hub1 overexpression explained 98% of transcriptional variance, indicating its dominant regulatory influence. Using rMATS, we detected seven exon skipping events, with *DYN2* showing significant differential splicing (FDR = 0.0481, ΔPSI = − 0.036). MaxEntScan analysis confirmed that *DYN2*’s 5′ splice site is significantly weaker than canonical yeast splice sites (score = − 18.32, *p* = 0.03), consistent with Hub1’s role in facilitating non-consensus splicing. Functional enrichment analyses revealed metabolic reprogramming, with upregulated pathways including biosynthesis of secondary metabolites and carbon metabolism, while growth-related processes like ribosome biogenesis and cell cycle were downregulated. Gene Set Enrichment Analysis (GSEA) further supported stress response activation (p53 signaling, NES = 1.255) and cell cycle suppression (NES = − 0.692). Weighted Gene Co-expression Network Analysis (WGCNA) identified 61 co-expression modules, with the brown module highly correlated with Hub1 overexpression (*r* = 0.99, *p* < 0.001) and enriched in biosynthetic and proteasome pathways. Protein-protein interaction network analysis revealed 35 Hub1 interactors, including spliceosomal components, reinforcing its central role in RNA processing.

**Conclusion:**

Our findings reveal that Hub1 overexpression drives coordinated transcriptional and post-transcriptional changes, promoting metabolic reprogramming while specifically modulating splicing of genes with weak splice sites like *DYN2*. These results establish Hub1 as a dual regulator linking transcriptional control with splicing precision, suggesting a regulatory mechanism that enhances cellular adaptability under stress conditions.

**Supplementary Information:**

The online version contains supplementary material available at 10.1186/s12864-025-12006-w.

## Introduction

Post-transcriptional regulation plays a pivotal role in shaping cellular responses to environmental and physiological stimuli [1]. A central mechanism within this regulatory layer is pre-mRNA splicing, which enables the generation of multiple transcript isoforms from a single gene, thereby expanding proteomic diversity and functional complexity [2]. This process is tightly regulated by various proteins, including a distinct class known as ubiquitin-like proteins (UBLs) [3]. Unlike classical ubiquitin, which typically mediates protein degradation via covalent modification, UBLs such as Hub1 (UBL5 in higher eukaryotes) exert their influence through non-covalent interactions, especially with components of the RNA processing machinery [4, 5].

Hub1 is an evolutionarily conserved UBL that modulates splicing fidelity and transcript maturation across eukaryotes [4]. In *Saccharomyces cerevisiae*, Hub1 enhances the splicing of pre-mRNAs with weak or non-consensus splice sites by interacting with spliceosomal proteins like Snu66 [6]. Although *S. cerevisiae* contains relatively few intron-bearing genes (~ 5%), many of these genes feature non-canonical splice sites, making them highly reliant on accessory factors like Hub1 [7]. Through this mechanism, Hub1 increases the efficiency and flexibility of the splicing machinery, facilitating the inclusion of otherwise poorly recognized exons [8]. Beyond its role in splicing, Hub1 has been implicated in diverse biological processes, including mitochondrial maintenance, transcriptional control, and cellular stress adaptation [9]. Studies have shown that Hub1 activity is modulated under stress conditions such as DNA damage and oxidative stress, suggesting that it may serve as a molecular bridge linking environmental sensing to RNA processing efficiency. This regulatory versatility makes Hub1 an important factor in maintaining cellular homeostasis [5].

Despite extensive characterization of its splicing-related functions, the transcriptome-wide effects of Hub1 overexpression remain incompletely understood. While previous studies have identified individual Hub1-regulated genes, a systems-level understanding of how elevated Hub1 levels reshape the expression and splicing landscape is still lacking [10]. Given the conservation of Hub1 across species and its emerging relevance in human disease contexts, such as splicing disorders and stress-related pathologies, elucidating its genome-wide regulatory influence in a model organism is both timely and important [4]. The budding yeast *S. cerevisiae* provides an ideal system for investigating these mechanisms. Its compact and well-annotated genome, coupled with low intron density and genetic tractability, allows for precise dissection of RNA processing events with minimal background complexity [11]. Additionally, the conservation of fundamental splicing components between yeast and humans ensures that insights gained from yeast studies can inform broader biological understanding [12].

In this study, we perform a comprehensive integrative analysis of publicly available RNA-seq data from *S. cerevisiae* strains overexpressing Hub1. Our pipeline incorporates multiple layers of transcriptomic analysis, including differential gene expression profiling, detection of alternative splicing events, splice site strength evaluation, functional enrichment, weighted gene co-expression network analysis (WGCNA), and protein-protein interaction (PPI) network construction [13–15]. This multifaceted approach enables a detailed assessment of both transcriptional and post-transcriptional changes induced by Hub1 overexpression. The analysis reveals that Hub1 drives widespread transcriptional reprogramming, significantly affecting genes involved in metabolic processes, ribosome biogenesis, and stress response [16, 17]. Simultaneously, Hub1 selectively modulates splicing events in a subset of genes characterized by weak splice sites such as *DYN2*, a gene involved in cytoskeletal organization and membrane dynamics [18]. Moreover, co-expression and PPI network analyses position Hub1 as a central regulatory node with connections to key pathways involved in RNA processing and cellular adaptation [19, 20]. Overall, this study positions Hub1 as a dual-function regulator that integrates transcriptional control with splicing precision. By coordinating these two regulatory layers, Hub1 enhances the cell’s ability to fine-tune gene expression programs in response to internal and external cues, underscoring its role in maintaining cellular homeostasis and adaptive capacity.

## Methods

### Data acquisition and quality assessment

RNA-seq data for *Saccharomyces cerevisiae* overexpressing Hub1 and wild-type controls were retrieved from the NCBI Gene Expression Omnibus (GEO) under accession number GSE84215 (accessed on July 5, 2025) [21]. The dataset comprised six paired-end RNA-seq samples: three biological replicates for the xrn1 rat1-1 control condition (SRR3822520, SRR3822521, SRR3822522) and three for the Hub1-overexpression condition (SRR3822526, SRR3822527, SRR3822528). Initial quality assessment was performed using FastQC v0.11.9 to evaluate read quality, adapter contamination, and base composition [22]. All samples passed quality thresholds with Phred scores > 30 for > 95% of bases and no significant adapter contamination, eliminating the need for additional preprocessing.

### Read alignment and processing

High-quality reads were aligned to the *Saccharomyces cerevisiae* reference genome (Ensembl R64-1-1, release 114) using HISAT2 v2.2.1with default parameters optimized for eukaryotic transcriptomes [23]. The HISAT2 index was constructed from the corresponding genome FASTA and GTF annotation files (*Saccharomyces_cerevisiae*.R64-1-1.114). Alignments were performed using the --dta (downstream transcriptome analysis) option to preserve alignment information necessary for transcript assembly and splicing analysis. The resulting SAM files were converted to BAM format, coordinate-sorted, and indexed using SAMtools v1.10 [24]. Alignment statistics were assessed to ensure > 85% mapping rates across all samples.

### Gene expression quantification and normalization

Gene-level read quantification was conducted using featureCounts from the Subread package v2.0.3 [25]. Parameters were set for paired-end mode (-p), unique mapping only (-Q 10), and strand-specific counting when applicable. The Ensembl GTF annotation file served as the gene model reference for read assignment. Resulting raw count matrices were filtered to remove genes with fewer than 10 total reads across all samples to reduce noise from lowly expressed transcripts. For transcript-level analysis, Salmon v1.10.1 was employed using the Ensembl R64-1-1 transcriptome FASTA to build the index [26]. Quantification was performed in paired-end quasi-mapping mode with the --validateMappings flag to improve alignment accuracy and --gcBias to correct for GC content bias. Output files containing transcript-level counts and TPM (Transcripts Per Million) values were imported into R using tximport v1.18.0 for downstream analysis [27].

### Differential gene expression analysis

Differential expression analysis was performed using DESeq2 v1.30.1 [13] in R v4.1.1. Raw count matrices were normalized using the median-of-ratios method to account for library size differences and compositional bias. Dispersion parameters were estimated using empirical Bayes shrinkage with the fitType="parametric” option. A negative binomial generalized linear model was fitted to model count data, and statistical significance was assessed using the Wald test. Multiple testing correction was applied using the Benjamini-Hochberg method, with genes considered significantly differentially expressed at adjusted p-value < 0.05 [28]. Log2 fold change shrinkage was applied using the apeglm method to reduce variance for lowly expressed genes. Principal component analysis (PCA) was performed on variance-stabilized transformed (VST) data using the top 500 most variable genes to assess sample clustering and batch effects. Visualization included volcano plots (log2 fold change vs. -log10 adjusted p-value) and MA plots (mean expression vs. log2 fold change) to evaluate the distribution and magnitude of expression changes [29].

### Alternative splicing event detection

Alternative splicing analysis was conducted using rMATS-turbo v4.1.2 [14], specifically designed for detecting differential splicing events from replicated RNA-seq data. Analysis parameters included paired-end reads (read length = 150 bp), minimum read coverage of 10 for inclusion/exclusion counts, and false discovery rate (FDR) threshold of 0.05. The analysis focused on five canonical splicing event types: skipped exons (SE), alternative 5’ splice sites (A5SS), alternative 3’ splice sites (A3SS), mutually exclusive exons (MXE), and retained introns (RI). Percent spliced-in (Ψ) values were calculated for each condition, and differential Ψ values (ΔΨ) quantified the magnitude of splicing regulation. Significant events were identified based on FDR < 0.05 and |ΔΨ| > 0.03. Sashimi plots were generated using rmats2sashimiplot to visualize junction read coverage and validate splicing events [30].

### Complementary exon usage analysis

To provide an alternative perspective on splicing regulation, differential exon usage analysis was performed using DEXSeq v1.48.0 [31]. This method models exon usage independently of predefined splicing events and can detect subtle changes in exon inclusion. The analysis used the same BAM files and GTF annotation, with significance determined at adjusted p-value < 0.05 after Benjamini-Hochberg correction.

### Splice site strength assessment

Splice site motif strength was evaluated using MaxEntScan to assess whether Hub1-regulated exons contained suboptimal splice sites [32]. Genomic coordinates for alternatively spliced exons were extracted, and flanking sequences were retrieved using BEDTools v2.30.0 [33]. Specifically, 9 bp sequences surrounding 5’ donor sites and 23 bp sequences around 3’ acceptor sites were extracted from the reference genome. These sequences were scored using MaxEntScan’s maximum entropy models (score5.pl and score3.pl), with lower scores indicating weaker splice site strength. Statistical comparison against canonical splice site distributions was performed using two-tailed t-tests [34].

### Functional enrichment analysis

Functional annotation of differentially expressed genes was performed using clusterProfiler v4.8.3 in R [35]. Gene identifiers were converted from Ensembl to Entrez format using the org.Sc.sgd.db annotation package. Over-representation analysis (ORA) was conducted for Gene Ontology (GO) terms across three domains: Biological Process (BP), Cellular Component (CC), and Molecular Function (MF), using the enrichGO() function. KEGG pathway enrichment was performed using enrichKEGG() with organism code ‘sce’ for *Saccharomyces cerevisiae*. Significance was determined at FDR-adjusted p-value < 0.05. Gene Set Enrichment Analysis (GSEA) was conducted using gseGO() and gseKEGG() functions on a pre-ranked gene list sorted by log2 fold change values from DESeq2 [36]. GSEA parameters included 1000 permutations and significance threshold of FDR < 0.2 to capture subtle but coordinated pathway-level changes [37].

### Weighted gene Co-expression network analysis

Co-expression network analysis was performed using WGCNA v1.71 to identify modules of co-regulated genes associated with Hub1 overexpression [15]. Low-variance genes below the 25th percentile was filtered out, and variance-stabilized counts from DESeq2 were used as input. A signed network was constructed with soft-thresholding power set to 12, determined by scale-free topology model fit criteria (R² > 0.8). Network construction and module detection were performed using the blockwiseModules() function with parameters: minimum module size = 30, deepSplit = 2, and mergeCutHeight = 0.25. Module eigengenes were calculated and correlated with the Hub1-overexpression trait using Pearson correlation. Modules with |correlation| > 0.8 and p-value < 0.05 were considered significantly associated with the experimental condition.

### Protein-Protein interaction network analysis

Protein-protein interaction networks were constructed using STRINGdb v2.10.0 to examine interaction patterns among Hub1 and differentially expressed genes [19]. Hub1 and its first-degree interactors were retrieved with a minimum combined confidence score of 400, encompassing experimental, database, and computational evidence. The resulting network was visualized using ggraph v2.0.5, with nodes colored by log2 fold change values to highlight expression patterns within the interaction network.

## Results

### Differential gene expression analysis

**Fig. 1:**
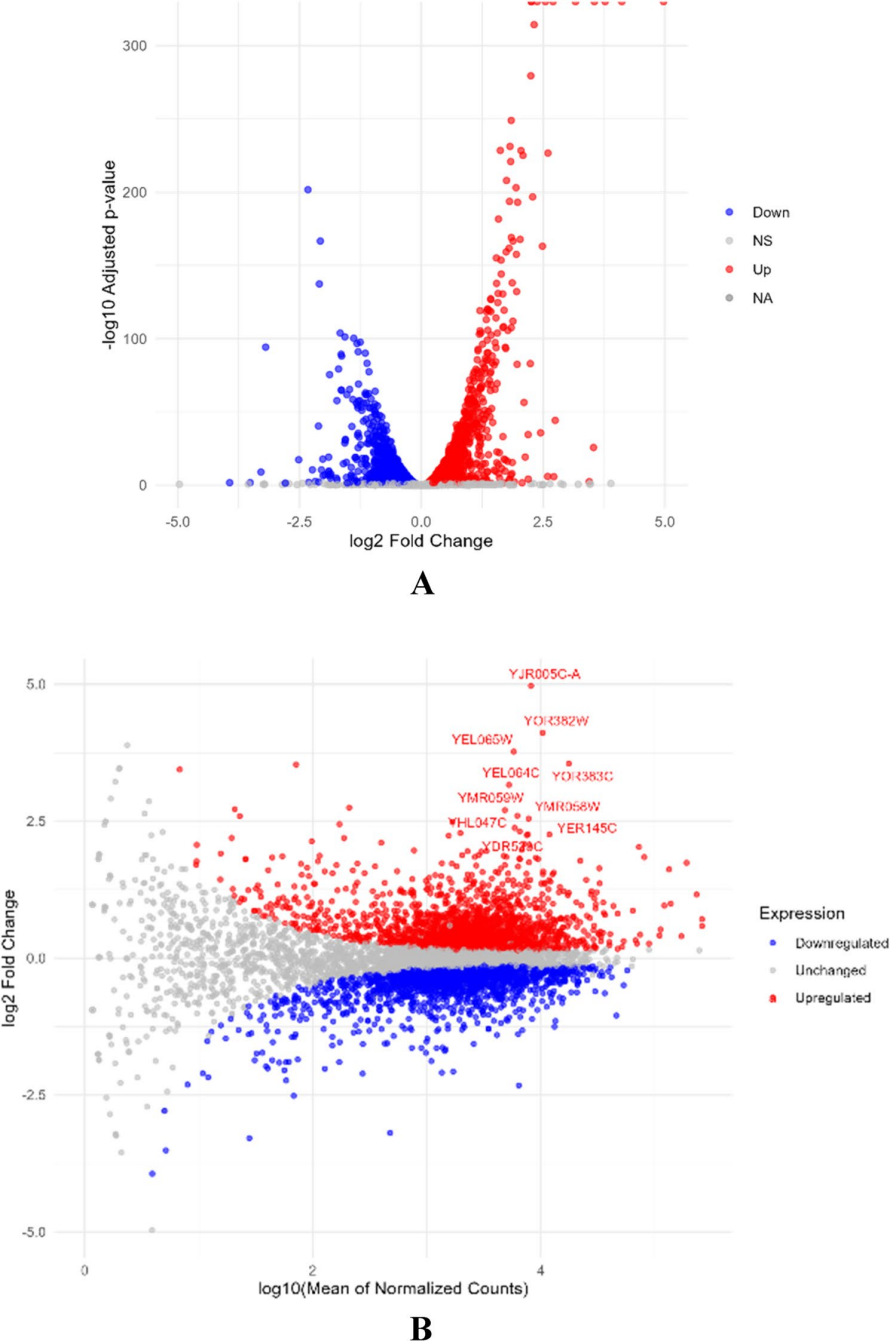
Differential gene expression analysis **A** Volcano plot showing differentially expressed genes between Hub1-overexpressing and control yeast strains. **B** MA plot of mean expression versus log2 fold change in Hub1-overexpressing yeast

To explore the global transcriptional response to Hub1 overexpression, RNA-seq data from three biological replicates per condition (Hub1-overexpressing vs. control) were analyzed using DESeq2. After normalization and statistical testing, a total of 3,915 genes were found to be differentially expressed with an adjusted p-value < 0.05. Of these, 1,964 genes were significantly upregulated, and 1,951 were downregulated, indicating a balanced but extensive transcriptional shift in response to Hub1. The volcano plot (Fig. 1A**)** visualizes the distribution of all genes based on statistical significance (–log10 adjusted p-value) and magnitude of expression change (log2 fold change). A substantial number of genes clustered at both ends of the x-axis demonstrate the strong upregulation (red dots) and downregulation (blue dots) of transcripts in the Hub1-overexpressing condition. Several of the most strongly regulated genes, such as YJR005C-A (log2FC = + 4.97), YOR382W (+ 4.11), and YOR383C (+ 3.55), appear in the upper-right quadrant of the plot, highlighting their elevated expression and statistical significance. On the opposite end, genes such as YHR124W and YGR250C displayed notable downregulation. To further examine gene expression trends relative to average abundance, an MA plot was constructed **(**Fig. 1B**).** This plot shows the relationship between mean expression (log counts per million) and log2 fold change for each gene. Significantly upregulated genes (in red) and downregulated genes (in blue) are symmetrically distributed around the zero-fold change line, confirming a global and balanced regulatory effect. Genes with high expression levels and extreme fold changes, such as YEL065W, YEL064C, and YMR058W, stand out as potential targets for further functional investigation. Together, these results suggest that Hub1 overexpression induces a strong and widespread effect on yeast gene expression, impacting both low- and high-abundance transcripts and implicating genes involved in RNA metabolism, stress response, and transcriptional regulation.

**Fig. 2:**
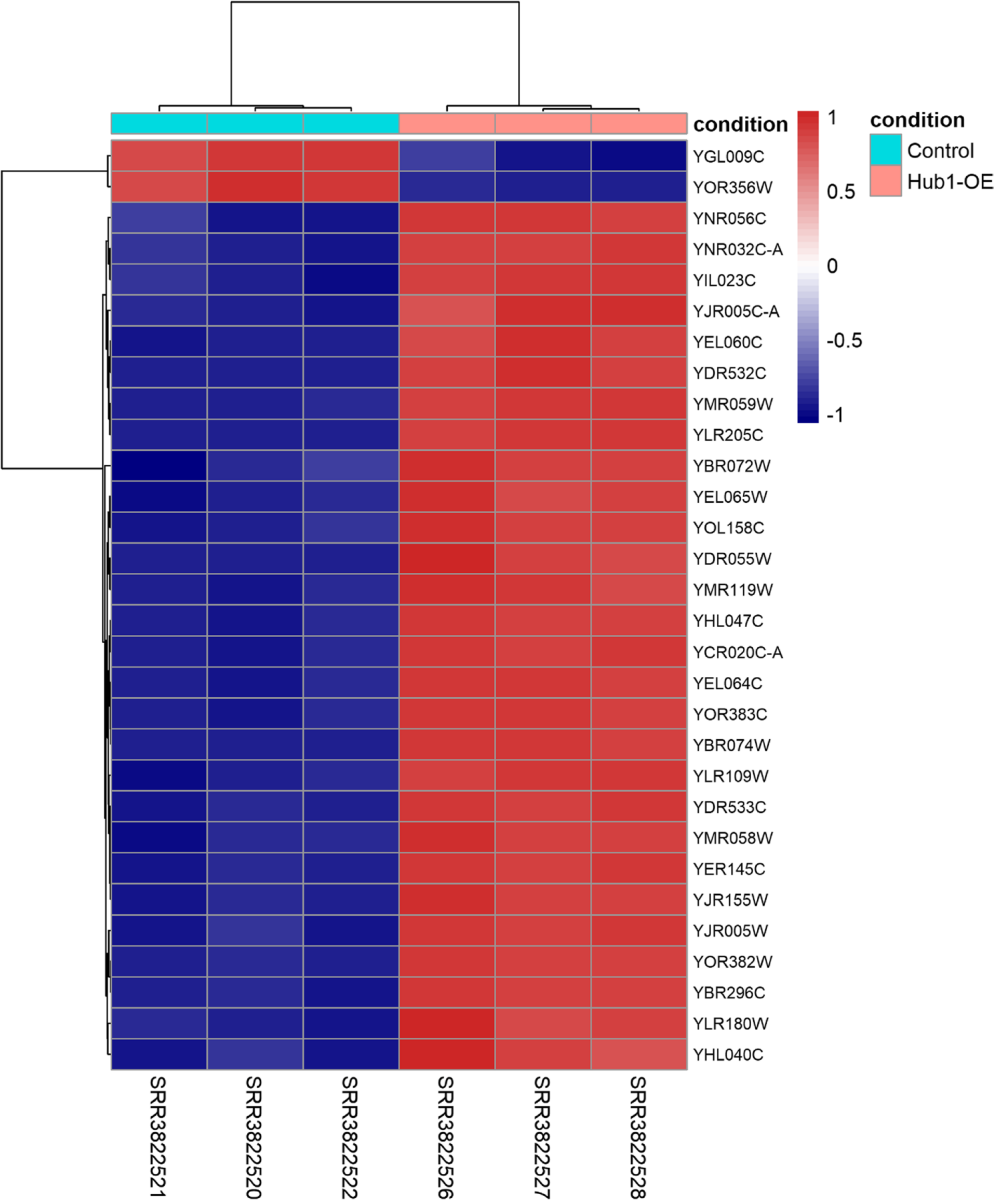
Heatmap of the top 30 differentially expressed genes in Hub1-OE and control samples. Each row represents a gene and each column a sample. Expression values were normalized and scaled (Z-scores). Genes were selected based on adjusted p-value and log₂ fold change from DESeq2 analysis

To highlight the most significant transcriptional changes, we listed the top 10 differentially expressed genes ranked by adjusted p-value and log2 fold change (Table 1). These genes showed substantial upregulation in Hub1-overexpressing samples compared to control, with many involved in RNA processing, stress response, and cell cycle regulation. The inclusion of genes such as YOR382W, YJR005C-A, and YMR059W underscores the strong transcriptional reprogramming driven by Hub1 overexpression. To further examine the expression dynamics of the most significantly regulated genes, a heatmap was constructed using the top 30 differentially expressed genes (DEGs), ranked by adjusted p-value and absolute log₂ fold change. Normalized expression values were transformed to Z-scores to highlight differential trends across samples. As shown in Fig. 2, hierarchical clustering revealed clear and consistent separation between Hub1-overexpressing and control groups. All three biological replicates per condition clustered tightly, confirming sample reproducibility. Genes upregulated in Hub1-OE samples exhibited coordinated increased expression, while downregulated genes were consistently suppressed in control samples. Notably, several genes from the top 10 DEG list (Table 1), including YJR005C-A, YOR382W, and YMR058W, showed strong upregulation. This reinforces the transcriptomic signature induced by Hub1 overexpression and supports the robustness of the differential expression analysis.

**Table 1:** Top 10 most significantly differentially expressed genes (DEGs) in Hub1-OE vs. control samples

Gene ID	log₂ Fold Change (log2FC)	Base Mean	Adjusted *p*-value (padj)*
YDR533C	+ 2.26	7,674	0
YOR382W	+ 4.11	10,351	0
YOR383C	+ 3.55	17,547	0
YMR059W	+ 2.70	4,820	0
YMR058W	+ 2.55	7,802	0
YJR005C-A	+ 4.97	8,186	0
YER145C	+ 2.26	11,851	0
YEL065W	+ 3.77	5,780	0
YEL064C	+ 3.16	5,241	0
YHL047C	+ 2.38	5,876	0

*Genes are ranked based on statistical significance (adjusted p-value) and magnitude of expression change

### Principle component analysis (PCA)

To evaluate the global transcriptional variance and assess sample clustering, principal component analysis (PCA) was conducted on variance-stabilized transformed (VST) gene expression data derived from the DESeq2 pipeline. The PCA plot (Fig. S1) demonstrates a clear separation between the Hub1-overexpressing (Hub1-OE) and control (xrn1 rat1-1) groups along the first principal component (PC1), which explains 98% of the total variance. The second principal component (PC2) accounts for only 1% of the variance, indicating that Hub1 overexpression is the predominant source of transcriptional variation. Biological replicates within each group clustered tightly, suggesting high experimental reproducibility and minimal batch or technical variability. The distinct separation observed along PC1 reinforces the conclusion that Hub1 overexpression induces a substantial and consistent shift in the yeast transcriptome. 

### Functional enrichment and pathway analysis of DEGs

**Fig. 3:**
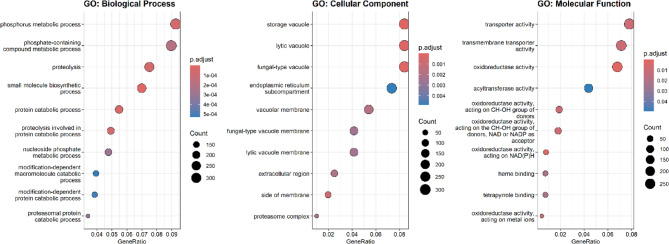
GO enrichment analysis of differentially expressed genes (DEGs) upon Hub1 overexpression in *Saccharomyces cerevisiae.*Dot plots display the top 10 significantly enriched GO terms for Biological Process (left), Cellular Component (middle), and Molecular Function (right)

To gain functional insights into the biological roles affected by Hub1 overexpression, we performed Gene Ontology (GO) and Kyoto Encyclopedia of Genes and Genomes (KEGG) enrichment analyses using differentially expressed genes (DEGs; adjusted p-value < 0.05). GO enrichment was conducted across three major domains: Biological Process (BP), Cellular Component (CC), and Molecular Function (MF). As shown in Fig. 3, the top enriched BP terms included proteolysis, phosphorus metabolic process, and small molecule biosynthetic process, indicating increased activity in protein degradation and metabolic regulation. In the CC category, enriched terms such as vacuolar membrane and endoplasmic reticulum subcompartment suggest that Hub1 influences membrane-associated organelle function. Within the MF domain, terms like oxidoreductase activity and transmembrane transporter activity were prominent, reflecting changes in redox biology and molecular transport mechanisms.

**Fig. 4:**
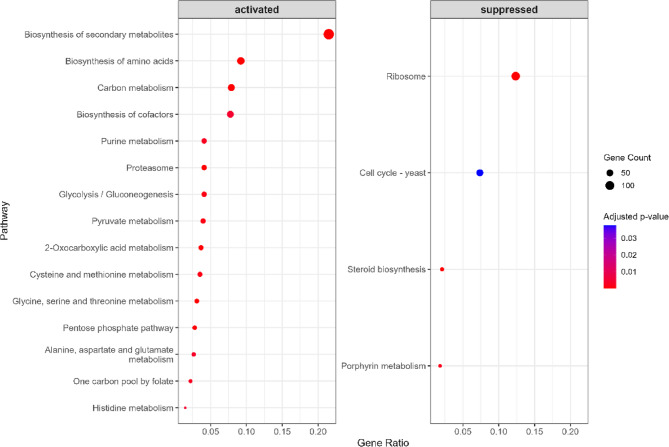
KEGG pathway enrichment analysis of DEGs in Hub1-overexpressing yeast. Dot plot showing the top 15 enriched KEGG pathways among upregulated (left) and downregulated (right) gene sets

To further explore pathway-level effects, KEGG pathway enrichment was performed separately for upregulated and downregulated genes using the clusterProfiler package [35]. As shown in Fig. 4, activated pathways in Hub1-OE samples included biosynthesis of secondary metabolites, *carbon metabolism*,* and purine metabolism*, indicating upregulation of biosynthetic and metabolic functions. Conversely, suppressed pathways such as ribosome, cell cycle - yeast, and steroid biosynthesis suggest downregulation of growth-related and translational machinery. These findings demonstrate that Hub1 overexpression induces a major transcriptomic shift favoring metabolic reprogramming while attenuating cell cycle and protein synthesis processes.

### Gene set enrichment analysis

**Fig. 5:**
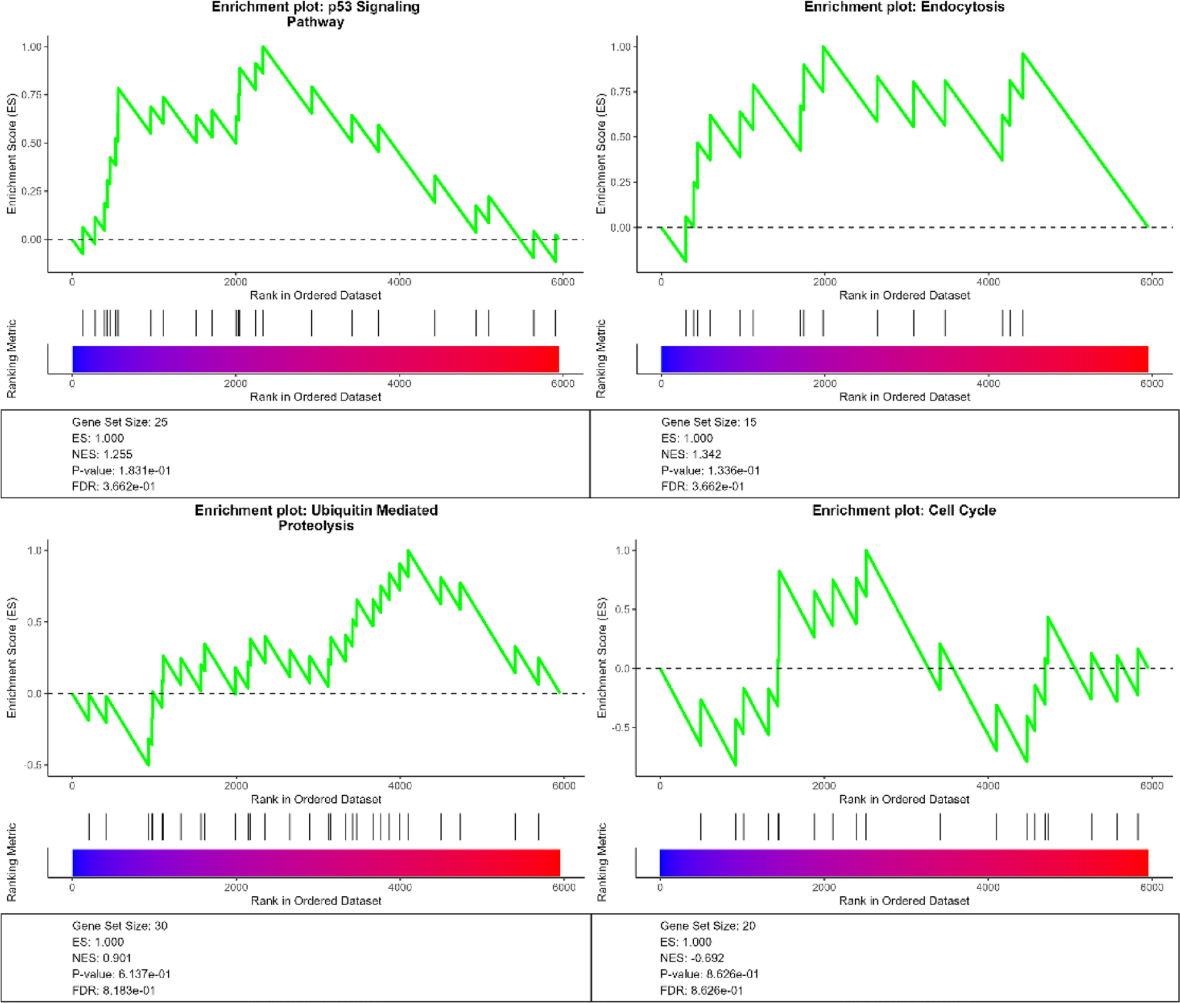
Gene Set Enrichment Analysis (GSEA) of four representative KEGG pathways under Hub1 overexpression.Each panel shows an enrichment plot for one KEGG pathway: **A** p53 signaling, **B** Endocytosis, **C** Ubiquitin-mediated proteolysis, and **D** Cell Cycle. Green lines indicate enrichment score (ES) curves calculated over the ranked log2 fold change–based gene list

To gain insights into the systemic effects of Hub1 overexpression, Gene Set Enrichment Analysis (GSEA) was performed using a ranked gene list based on DESeq2-derived log2 fold changes. This analysis aimed to identify whether predefined gene sets (KEGG pathways) were enriched among the genes most strongly affected by Hub1 overexpression. As shown in Fig. 5, several biologically relevant pathways exhibited notable enrichment patterns. The p53 signaling pathway and Endocytosis were modestly enriched in upregulated genes, with normalized enrichment scores (NES) of 1.255 and 1.342, respectively, suggesting an association with stress response and membrane trafficking. Ubiquitin-mediated proteolysis showed a nearly neutral enrichment (NES = 0.001), while the Cell Cycle pathway displayed a modest negative enrichment (NES = − 0.692), indicating potential transcriptional repression of cell division–related genes under Hub1-OE. These enrichment dynamics support the hypothesis that Hub1 modulates global transcriptional programs, including proteostasis, stress signaling, and cell cycle control, thereby reshaping the physiological state of *Saccharomyces cerevisiae*.

### Alternative splicing analysis

**Fig. 6:**
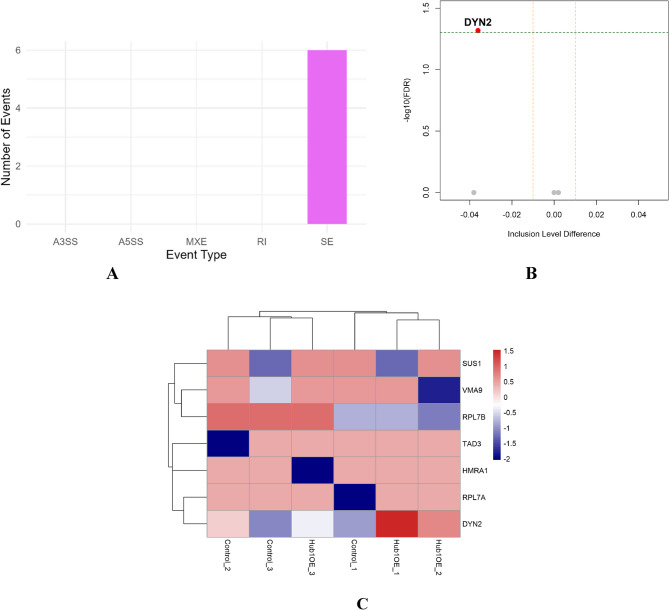
Alternative splicing analysis using rMATS identifies a single significant SE event. **A** Bar plot showing the number of detected events across five alternative splicing types. **B** Volcano plot of SE events comparing Hub1-OE to control conditions. The DYN2 gene was the only event surpassing the significance threshold (FDR < 0.05, |ΔΨ| > 0.03), indicating reduced exon inclusion. **C** Heatmap of Z-score normalized exon inclusion levels for all SE events. Samples cluster by condition, with DYN2, RPL7A, and SUS1 showing notable splicing differences.

To assess the impact of Hub1 overexpression on pre-mRNA splicing, we performed a comprehensive analysis using rMATS v4.1.2, comparing three biological replicates of Hub1-overexpressing (*Hub1-OE*) yeast to three controls. Among the five canonical types of alternative splicing (AS) events, only exon skipping (SE) events were detected in the *Saccharomyces cerevisiae* transcriptome (Fig. 6A). A total of seven SE events were identified, consistent with the known low splicing complexity in yeast, where ~ 95% of genes lack introns. No events were detected for alternative 3′ or 5′ splice sites (A3SS, A5SS), mutually exclusive exons (MXE), or retained introns (RI), indicating that Hub1 may selectively modulate exon skipping under the tested conditions. To identify significantly regulated AS events, we constructed a volcano plot based on inclusion level difference (ΔΨ) and false discovery rate (FDR). Among the seven SE events, only one gene *DYN2* exhibited statistically significant differential splicing with FDR = 0.0481 and ΔΨ = − 0.036 (Fig. 6B). This suggests a modest reduction in exon inclusion in the *Hub1-OE* condition and a potential target-specific role of Hub1 in modulating splicing precision.

**Fig. 7:**
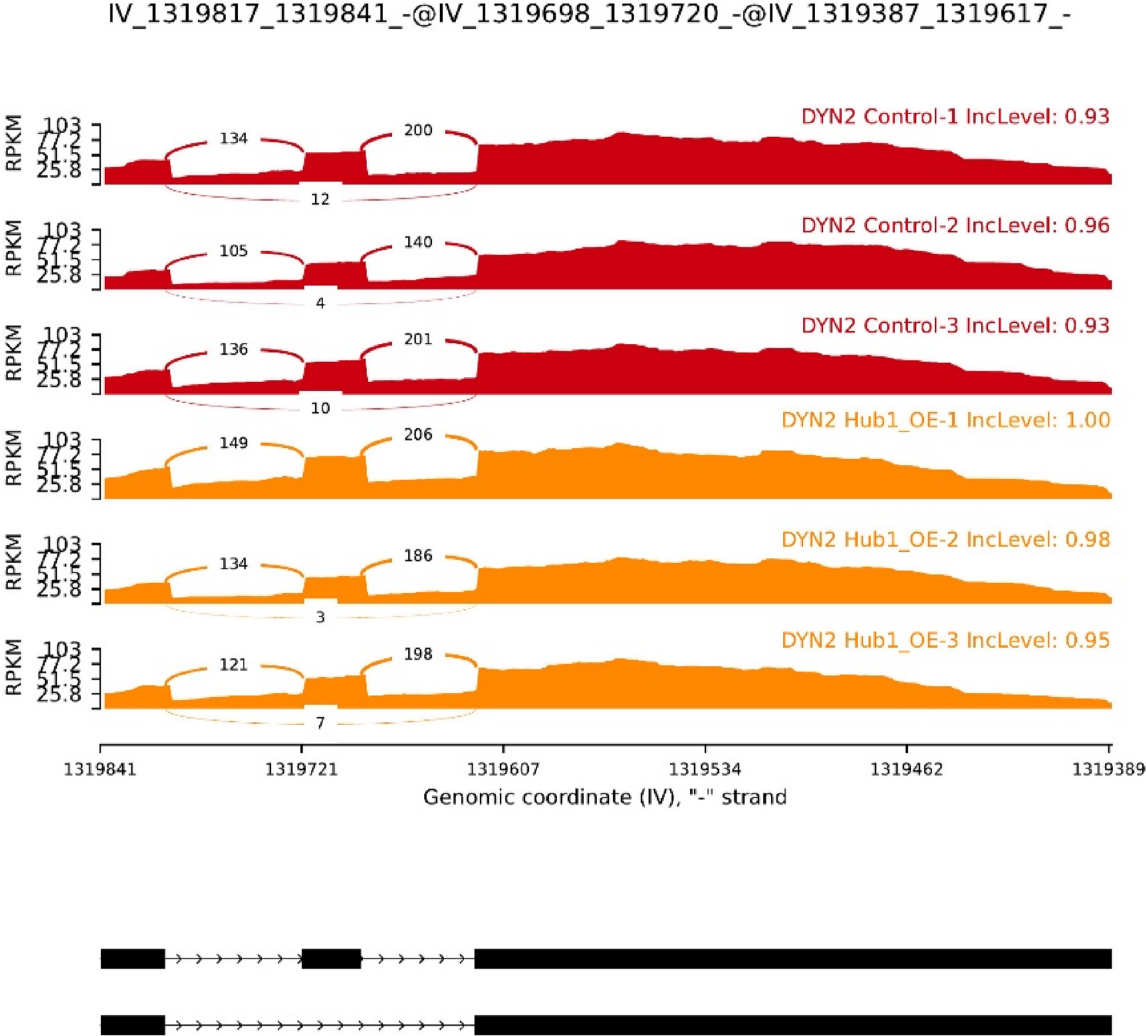
Sashimi plot validating DYN2 exon skipping event in Hub1-overexpressing yeast.Exon junction usage in *DYN2* was visualized using sashimi plots generated from BAM files. Read density and junction arcs indicate inclusion of the alternatively spliced exon. *Hub1-OE* samples show slightly increased exon inclusion (~95–100%) compared to controls (~93–96%), consistent with rMATS output (ΔΨ = –0.036; FDR = 0.0481)

We next visualized exon inclusion patterns across all SE events using a Z-score normalized heatmap (Fig. 6C). The heatmap revealed clear sample clustering by condition, with replicates from *Hub1-OE* and control groups forming distinct groups. While *DYN2* was the only statistically significant event, other genes such as *RPL7A* and *SUS1* also showed consistent exon usage differences, hinting at subtle splicing modulation. To validate the splicing alteration in *DYN2*, we generated a sashimi plot depicting exon junction read coverage and inclusion levels across samples (Fig. 7). The exon displayed high inclusion in both groups (93–96% in controls vs. 95–100% in *Hub1-OE*), supporting the rMATS prediction despite the small ΔΨ value. Although the change is modest, the consistent pattern and statistical support suggest a reproducible splicing effect associated with Hub1 activity.

### Exon usage analysis

To evaluate alternative splicing events modulated by Hub1 overexpression, we performed a comprehensive exon usage analysis using DEXSeq. This revealed a distinct pattern of differential exon regulation between Hub1-OE and control yeast strains. The MA plot (Fig. S2A) illustrates the global distribution of exon usage changes. A total of 27 exons were significantly downregulated and 21 exons upregulated (adjusted p-value < 0.05), while 970 exons showed no significant change. Noteworthy among the top differentially spliced regions were mitochondrial genome loci (Q0050 + Q0060 + Q0065 + Q0070 + Q0045 + Q0055), ribosomal subunits (YNL112W, YBR116C + YBR117C), and overlapping non-coding ORFs (YPR160W-A + YPR160W), implicating Hub1 in the regulation of both energy metabolism and ribosome assembly at the splicing level. The hierarchical clustering heatmap (Fig. S2B) of normalized exon counts confirmed consistent and condition-specific patterns across biological replicates. Exons such as E002 and E004 showed upregulation in Hub1-OE samples, while E003 and E001 were downregulated, suggesting a coordinated shift in exon inclusion or exclusion. Together, these findings support the hypothesis that Hub1 overexpression influences splicing fidelity and targets specific exon regions, especially in mitochondrial and ribosomal gene contexts.

### Weighted gene co-expression network analysis

**Fig. 8:**
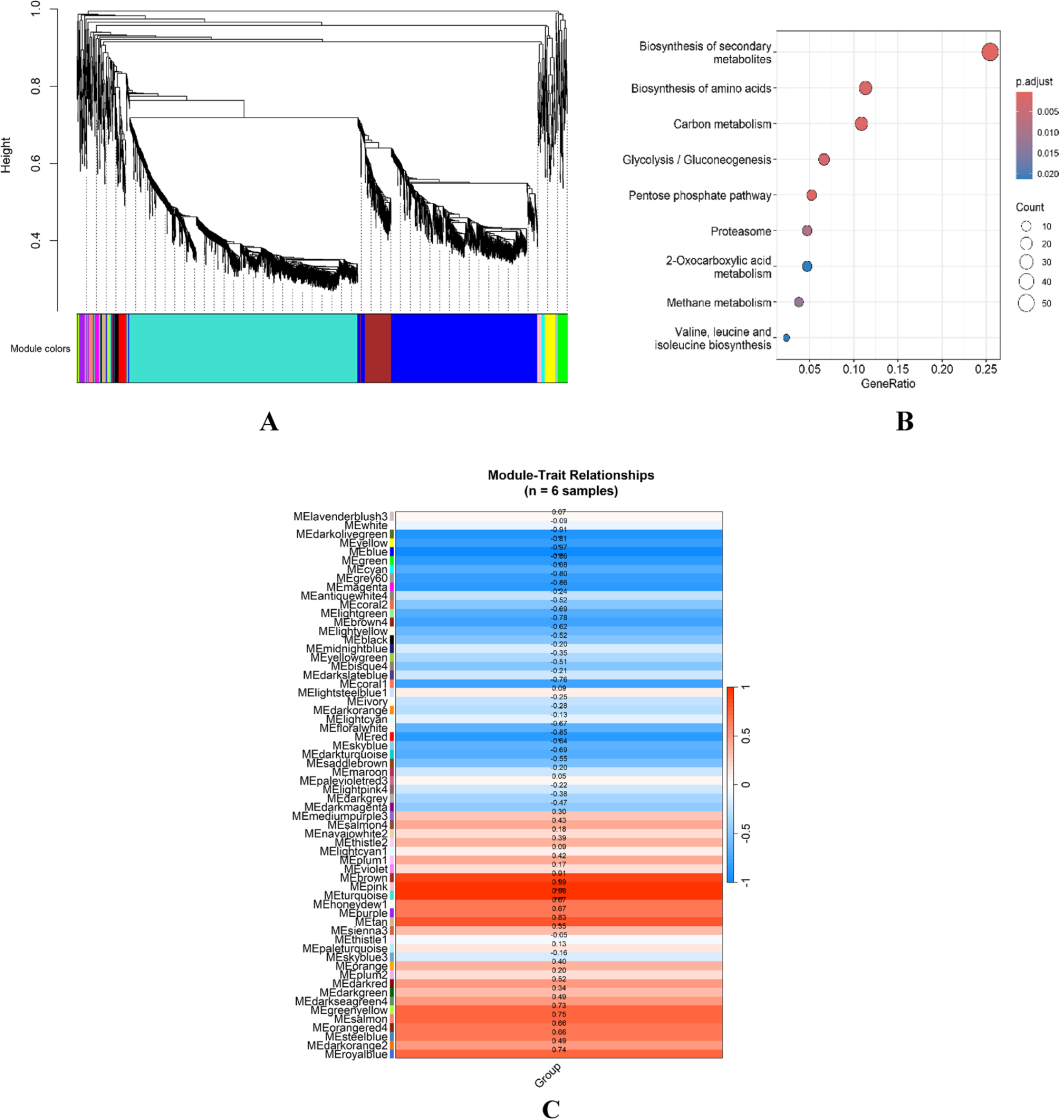
Weighted gene co-expression network analysis (WGCNA) of Hub1-OE transcriptome. **A** Cluster dendrogram of genes based on topological overlap matrix (TOM). Each leaf represents a gene, and branches are grouped into co-expression modules labeled by unique colors. **B** Heatmap of Pearson correlation coefficients between module eigengenes and experimental trait (Hub1 overexpression vs. control). Red and blue indicate positive and negative correlations, respectively. **C** KEGG pathway enrichment dot plot for the brown module. Dot size reflects the number of genes; color intensity indicates adjusted*p*-value. Top enriched pathways include biosynthetic and proteasome-related functions.

To explore transcriptional co-regulation in response to Hub1 overexpression, we performed Weighted Gene Co-expression Network Analysis (WGCNA) on the filtered expression matrix of 2,178 genes across six RNA-seq samples. After removing low-variance genes, a soft-thresholding power of 12 was selected to construct a signed scale-free network. Hierarchical clustering based on the topological overlap matrix (TOM) identified 61 co-expression modules, each assigned a unique color label (Fig. 8A). To assess the relationship between gene modules and experimental conditions, we correlated module eigengenes with a binary trait variable distinguishing Hub1-overexpressing and control samples. The module–trait relationship heatmap revealed several modules with significant correlations (Fig. 8B). Notably, the brown module showed the strongest positive association with Hub1 overexpression (*r* = 0.99, *p* < 0.001), followed by the pink (*r* = 0.98, *p* < 0.001) and turquoise (*r* = 0.83, *p* < 0.01) modules. Conversely, the darkolivegreen (*r* = − 0.91, *p* < 0.001) and yellow (*r* = − 0.81, *p* < 0.001) modules exhibited strong negative correlations, indicating potential downregulation of associated gene networks. To uncover functional insights, we performed KEGG pathway enrichment analysis on genes from the brown module, which was most strongly associated with Hub1 overexpression. The enriched pathways included biosynthesis of secondary metabolites, biosynthesis of amino acids, proteasome, and carbon metabolism (adjusted *p* < 0.01), reflecting a transcriptional shift toward enhanced metabolic and proteolytic activity (Fig. 8C). These results suggest that Hub1 overexpression may promote biosynthetic and growth-related programs while repressing mitochondrial energy production, consistent with the observed suppression of oxidative phosphorylation pathways in negatively correlated modules.

### Isoform-level quantification analysis

**Fig. 9:**
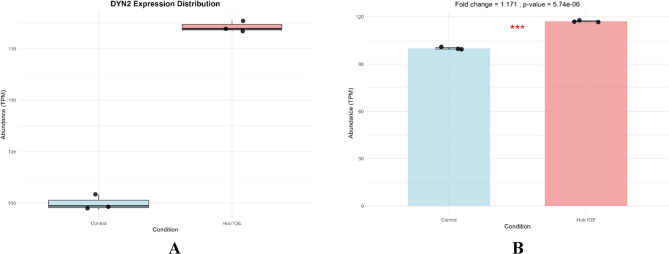
*DYN2* is significantly upregulated in response to Hub1 overexpression.** A** Bar plot of DYN2 transcript abundance (TPM) in control vs. Hub1OE samples. Error bars indicate standard deviation (n = 3). **B** Box plot of DYN2 TPM values across replicates. Tight clustering of values indicates high technical reproducibility. Asterisks (***) indicate statistical significance at p < 0.001

To explore isoform-specific expression changes associated with Hub1 overexpression, transcript-level quantification was performed using Salmon and normalized to TPM values. Among the analyzed transcripts, DYN2 (YDL007W) a gene encoding a dynamin-like GTPase involved in microtubule organization and endocytosis was found to be significantly upregulated. As shown in Fig.9, the mean TPM of DYN2 increased from 100.0 ± 0.8 in control samples to 117.1 ± 0.5 in Hub1-overexpressing (Hub1OE) samples, corresponding to a 17.1% increase (log₂FC = 0.228). This difference was statistically significant (p = 5.74 × 10⁻⁶, two-tailed unpaired t-test), indicating robust transcriptional activation of DYN2 under Hub1-OE conditions. Both the bar plot (Fig.9A) and box plot (Fig.9B) illustrate the consistent upregulation with low intra-group variability (CV < 1%). These results suggest a functional link between Hub1 expression and DYN2 transcriptional regulation, potentially implicating Hub1 in pathways related to cytoskeletal dynamics and membrane trafficking.

### Splice site motif analysis

**Fig. 10:**
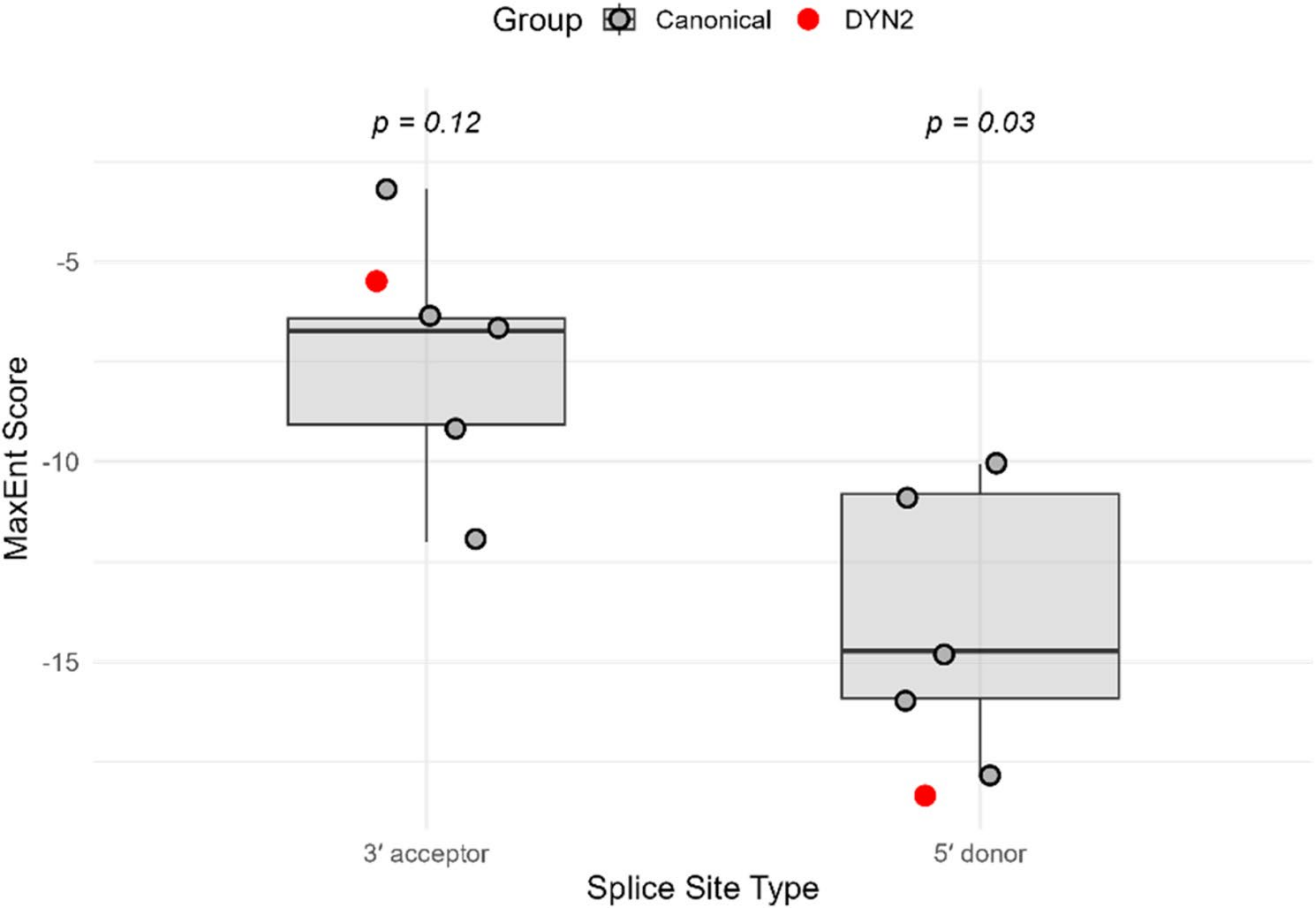
MaxEntScan splice site strength scores for Hub1-regulated exons**.** Boxplots show distributions of canonical yeast 5′ donor and 3′ acceptor splice site scores (gray). Red dots indicate the DYN2 exon’s donor (−18.32) and acceptor (−5.52) scores

To investigate whether Hub1 preferentially regulates exons with weak splice site signals, we performed motif strength analysis using MaxEntScan on differentially used exons, including the Hub1-responsive DYN2 exon. Splice site sequences (9 bp for 5′ donor, 23 bp for 3′ acceptor) were extracted from the *Saccharomyces cerevisiae* genome and evaluated using MaxEntScan scoring models. The 5′ donor site of the DYN2 exon scored − 18.32, which was significantly lower than the average canonical 5′ donor site score (mean = − 13.88, SD = 3.20), with a p-value of 0.03 (Fig. 10). This suggests that the DYN2 exon has a notably weak donor site. The 3′ acceptor site of DYN2 scored − 5.52, also weaker than canonical scores (mean = − 7.88, SD = 3.15), though this difference did not reach statistical significance (*p* = 0.12). In addition to DYN2, we evaluated five other Hub1-regulated exons (YDL246C_E1, YDL243C_E1, YDR387C_E1, YDL094C_E1, and YDR438W_E1). All exhibited low MaxEnt scores at either the donor or acceptor sites (Table S1). These findings support a model in which Hub1 facilitates the recognition of non-consensus or suboptimal splice sites, consistent with its known role in modulating spliceosome flexibility.

### Protein- protein interaction analysis

**Fig. 11:**
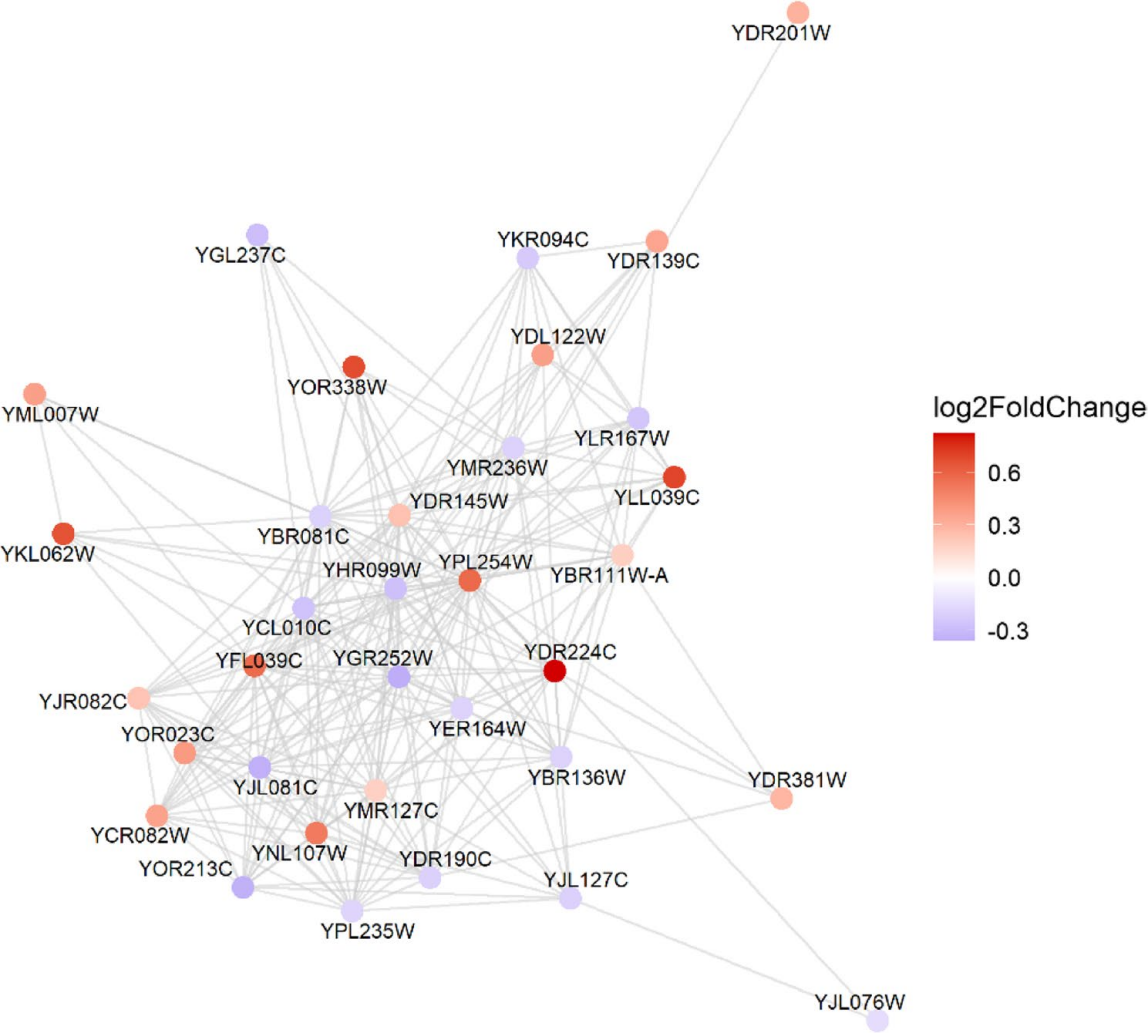
Protein-protein interaction (PPI) network of Hub1 (YPL254W) and its first-degree interactors. The network displays Hub1 (YPL254W) at the center along with 35 first-degree interacting proteins identified from the STRING database (combined score > 400). Nodes represent proteins, and edges indicate predicted interactions based on experimental and computational evidence. Node color reflects log₂ fold change in expression between Hub1-overexpressing and control cells, with red indicating higher expression and blue indicating lower expression

To explore the broader regulatory context of Hub1 (YPL254W), we constructed a protein–protein interaction (PPI) network using high-confidence STRING interactions (combined score > 400). This network focused on first-degree interactors of Hub1 and incorporated expression data from our RNA-seq analysis. The resulting subnetwork revealed 35 direct interactors (Fig. 11), many of which are differentially expressed under Hub1 overexpression. Notably, YDR224C and YLL039C were among the top upregulated interactors, with log₂ fold changes of + 0.69 and + 0.65, respectively, while YPL235W and YJL076W showed modest downregulation (log₂FC = − 0.33 and − 0.31). Several interactors, including YBR111W-A (a homolog of SNU66) and YHR099W (a homolog of SPP381), are known components of the spliceosomal machinery, reinforcing Hub1’s proposed role in splicing fidelity and exon definition. In addition, interactors like YDR145W, a chromatin remodeling factor, suggest a multi-layered role of Hub1 in transcriptional and post-transcriptional gene regulation. These findings highlight that Hub1 operates within a functionally coherent network, potentially linking RNA processing with chromatin state, and support a mechanistic model where Hub1 modulates non-consensus splicing through interaction with key spliceosomal and regulatory proteins.

## Discussion

The integrative transcriptomic and splicing analysis presented in this study elucidates the multifaceted role of Hub1 overexpression in *Saccharomyces cerevisiae*, revealing its profound impact on both gene expression and alternative splicing. By leveraging RNA-seq data from the GSE84215 dataset and employing a comprehensive bioinformatics pipeline, we identified 3,915 differentially expressed genes (DEGs) and seven exon skipping events, with a significant alteration in the *DYN2* gene (FDR = 0.0481, ΔPSI = − 0.036) **(**Figs. 6B and 7**)**. These findings underscore Hub1’s dual role as a regulator of transcriptional reprogramming and splicing fidelity, particularly in modulating non-consensus splice sites, and provide novel insights into its contributions to cellular homeostasis and stress adaptation.

The extensive transcriptional changes observed, with 1,964 upregulated and 1,951 downregulated genes (Fig. 1A; Table 1), highlight Hub1’s capacity to orchestrate a balanced yet widespread shift in the yeast transcriptome. The top differentially expressed genes, such as YJR005C-A (log2FC = + 4.97) and YOR382W (log2FC = + 4.11) **(**Table 1), are implicated in RNA processing and stress response, aligning with Hub1’s known functions in RNA metabolism and cellular adaptation [16]. The principal component analysis further confirmed the robustness of these changes, with PC1 explaining 98% of the transcriptional variance and clearly separating Hub1-overexpressing samples from controls (Fig. S1). This suggests that Hub1 overexpression is a dominant driver of global gene expression dynamics, consistent with its role as a ubiquitin-like modifier that interacts non-covalently with regulatory complexes [38]. The heatmap analysis of the top 30 differentially expressed genes (Fig. 2) demonstrated clear hierarchical clustering between experimental conditions, with all biological replicates showing consistent expression patterns. This reproducibility across replicates strengthens confidence in the observed transcriptional signature and supports the conclusion that Hub1 overexpression induces systematic changes in gene expression rather than stochastic effects.

Functional enrichment analyses provided deeper insights into the biological processes modulated by Hub1. Gene Ontology analysis revealed enriched terms such as proteolysis, phosphorus metabolic process, and vacuolar membrane function (Fig. 3), indicating that Hub1 influences protein degradation and organelle-associated processes. Similarly, KEGG pathway enrichment highlighted the upregulation of biosynthetic pathways (e.g., secondary metabolites, carbon metabolism) and downregulation of growth-related pathways (e.g., ribosome, cell cycle) **(**Fig. 4). These findings suggest that Hub1 overexpression induces metabolic reprogramming, potentially redirecting cellular resources toward stress response and biosynthetic activity while attenuating proliferation. Gene Set Enrichment Analysis (GSEA) further supported this metabolic shift, with modest enrichment of stress-related pathways like p53 signaling (NES = 1.255) and endocytosis (NES = 1.342), alongside negative enrichment of cell cycle genes (NES = − 0.692) (Fig. 5). These patterns align with prior reports linking Hub1 to stress adaptation and proteostasis [9, 38], and they suggest a coordinated regulatory program that balances cellular growth and survival under altered Hub1 levels. The downregulation of ribosome biogenesis and cell cycle pathways, combined with upregulation of stress response mechanisms, indicates that Hub1 overexpression may trigger a cellular state reminiscent of environmental stress adaptation [39].

The alternative splicing analysis revealed a targeted effect of Hub1 on exon skipping, with *DYN2* emerging as the only statistically significant event (FDR = 0.0481, ΔPSI = − 0.036) among seven detected exon skipping events (Fig. 6A and B). The modest reduction in *DYN2* exon inclusion, validated by sashimi plots (Fig. 7), underscores Hub1’s role in modulating splicing precision, particularly for genes with weak splice sites [40]. MaxEntScan analysis confirmed that the 5′ splice site of the *DYN2* exon is significantly weaker than canonical yeast splice sites (score = − 18.32, *p* = 0.03) (Fig. 10), consistent with Hub1’s established function in enhancing non-consensus splice site recognition [6]. The complementary DEXSeq analysis identified 48 differentially used exons (Figure S2A), with hierarchical clustering revealing consistent condition-specific patterns (Fig. S2B). Notably, several mitochondrial genome loci and ribosomal subunits showed differential exon usage, suggesting that Hub1’s splicing effects extend beyond nuclear genes to organellar transcripts. This broader splicing impact aligns with Hub1’s known role in facilitating splicing of genes with suboptimal splice sites [10]. The splice site strength analysis of Hub1-regulated exons (Table S1) revealed that most targeted exons contain weak donor or acceptor sites, supporting a model where Hub1 facilitates splicing of suboptimal sites through interactions with spliceosomal components like Snu66 [41]. Given that only ~ 5% of *S. cerevisiae* genes contain introns [42], the detection of even a small number of splicing events is notable and suggests that Hub1’s splicing role is highly selective yet functionally important. The significant splicing alteration in *DYN2*, a gene encoding a dynamin-like GTPase involved in microtubule organization and endocytosis [43], points to a functional link between Hub1 activity and cytoskeletal dynamics. The observed 17.1% increase in *DYN2* transcript abundance (log2FC = 0.228, *p* = 5.74 × 10⁻⁶) **(**Fig. 9**)** suggests that Hub1 not only modulates *DYN2* splicing but also enhances its transcriptional output. This dual regulation may amplify *DYN2*’s role in membrane trafficking and cytoskeletal remodeling, processes critical for stress adaptation and cellular homeostasis [44]. The enrichment of endocytosis pathways in GSEA (Fig. 5) further supports this connection, suggesting that Hub1 may coordinate splicing and expression changes to enhance endocytic pathways under stress conditions.

Weighted Gene Co-expression Network Analysis (WGCNA) provided a systems-level perspective on Hub1’s regulatory influence, identifying 61 co-expression modules, with the brown module showing the strongest correlation with Hub1 overexpression (*r* = 0.99, *p* < 0.001) (Fig. 8B). This module’s enrichment in biosynthetic and proteasome-related pathways (Fig. 8C) reinforces the notion that Hub1 drives metabolic and proteolytic reprogramming. The negative correlation of modules like darkolivegreen and yellow with Hub1 overexpression suggests a coordinated suppression of specific cellular programs, potentially including mitochondrial energy production as part of a broader stress response strategy [6]. The tight clustering of genes within co-expression modules (Fig. 8A) and their strong correlation with experimental conditions demonstrate that Hub1 overexpression affects not just individual genes but entire regulatory networks [45]. This systems-level impact underscores Hub1’s role as a master regulator capable of orchestrating complex transcriptional programs [46]. The protein-protein interaction (PPI) network analysis illuminated Hub1’s regulatory context, identifying 35 direct interactors, including spliceosomal components like YBR111W-A (SNU66 homolog) and YHR099W (SPP381 homolog) (Fig. 11). The differential expression of these interactors, such as the upregulation of YDR224C (log2FC = + 0.69) and YLL039C (log2FC = + 0.65), suggests that Hub1 operates within a functionally coherent network that integrates splicing and transcriptional regulation [6]. The presence of chromatin remodeling factors among the interactors further indicates that Hub1’s effects may extend to epigenetic regulation, consistent with its reported roles in nuclear envelope maintenance and chromatin remodeling [47].

This multi-layered regulatory network underscores Hub1’s versatility as a ubiquitin-like modifier and its capacity to bridge post-transcriptional and transcriptional control [5]. The coordinated expression changes among Hub1 interactors suggest that Hub1 overexpression may amplify its regulatory effects through positive feedback loops within its interaction network. The findings of this study have significant implications for understanding Hub1’s role in eukaryotic gene regulation. The conservation of Hub1 across species suggests that its regulatory mechanisms in *S. cerevisiae* may inform studies in higher eukaryotes, where Hub1 (UBL5) is implicated in splicing and stress response [48]. The specific modulation of *DYN2* splicing and expression highlights a potential regulatory axis linking Hub1 to cytoskeletal dynamics and stress adaptation, which warrants further investigation in other model systems. The metabolic reprogramming observed under Hub1 overexpression, characterized by enhanced biosynthetic activity and reduced growth-related processes, suggests that Hub1 may function as a molecular switch that adjusts cellular priorities in response to stress or environmental changes [49, 50]. This regulatory mechanism could be particularly important in organisms facing fluctuating environmental conditions, where rapid adjustment of gene expression programs is crucial for survival [51].

While this study provides comprehensive insights into Hub1’s regulatory role, several limitations must be acknowledged. The analysis relies on a single experimental dataset, potentially limiting the generalizability of findings across different growth conditions or stress scenarios [52]. The lack of experimental validation through RT-PCR or functional assays represents a significant limitation [53]. Future studies should prioritize experimental confirmation of the identified splicing changes and functional characterization of their cellular consequences [54]. Additionally, investigation of Hub1’s regulatory mechanisms under diverse stress conditions would provide deeper insights into its adaptive functions [55]. The modest effect size of the *DYN2* splicing change, while statistically significant, raises questions about biological relevance that can only be addressed through functional validation [56]. Complementation studies using splicing-defective *DYN2* variants could determine whether subtle splicing changes translate to measurable phenotypic effects.

## Conclusion

This study establishes Hub1 as a dual regulator of transcriptional reprogramming and alternative splicing in *Saccharomyces cerevisiae*, coordinating metabolic adaptation and stress response through integrated regulatory mechanisms. The extensive transcriptional changes (3,915 DEGs) coupled with selective splicing modulation demonstrate Hub1’s capacity to orchestrate complex cellular reprogramming while maintaining homeostatic balance. The identification of *DYN2* as a primary splicing target, characterized by weak splice sites and dual transcriptional-splicing regulation, exemplifies Hub1’s role in expanding regulatory precision through multiple control layers. The systems-level analysis reveals Hub1’s integration within highly correlated gene networks and protein interaction complexes, positioning it as a central node in cellular regulatory hierarchies. These findings advance our understanding of ubiquitin-like protein function beyond traditional protein modification roles, revealing Hub1’s capacity to integrate transcriptional and post-transcriptional regulation. The conservation of Hub1 across eukaryotes suggests that these regulatory mechanisms may be fundamental to cellular adaptation strategies, with potential implications for understanding disease processes involving splicing dysregulation.

## Supplementary Information

Additional file 1: Supplementary Figures and Tables. Contains supplementary figures S1-S2 and supplementary table S1 with detailed legends and methodological descriptions.

## Data Availability

The RNA-seq data that support the findings of this study are publicly available in the NCBI Gene Expression Omnibus (GEO) under accession number GSE84215 at https://www.ncbi.nlm.nih.gov/geo/query/acc.cgi? acc=GSE84215. The associated BioProject, SRA, and BioSample identifiers are PRJNA328390, SRP078212, and GSM2229494 to GSM2229496, respectively.

## References

[1] Van Assche E, Van Puyvelde S, Vanderleyden J, Steenackers HP. RNA-binding proteins involved in post-transcriptional regulation in bacteria. Front Microbiol. 2015;6:141. 25784899 10.3389/fmicb.2015.00141 PMC4347634

[2] Wang Y, Liu J, Huang BO, Xu YM, Li J, Huang LF, et al. Mechanism of alternative splicing and its regulation. Biomed Rep. 2015;3(2):152–8. 25798239 10.3892/br.2014.407 PMC4360811

[3] Bedford L, Lowe J, Dick LR, Mayer RJ, Brownell JE. Ubiquitin-like protein conjugation and the ubiquitin-proteasome system as drug targets. Nat Rev Drug Discov. 2011;10(1):29–46. 21151032 10.1038/nrd3321 PMC7097807

[4] Kolathur KK, Sharma P, Kadam NY, Shahi N, Nishitha A, Babu K, et al. The ubiquitin-like protein Hub1/UBL-5 functions in pre-mRNA splicing in caenorhabditis elegans. FEBS Lett. 2023;597(3):448–57. 36480405 10.1002/1873-3468.14555 PMC7615767

[5] Chanarat S. UBL5/Hub1: an atypical Ubiquitin-Like protein with a typical role as a Stress-Responsive regulator. Int J Mol Sci. 2021;22(17):9384–4. 10.3390/ijms22179384 PMC8431670 34502293

[6] Mishra SK, Ammon T, Popowicz GM, Krajewski M, Nagel RJ, Ares M Jr., et al. Role of the ubiquitin-like protein Hub1 in splice-site usage and alternative splicing. Nature. 2011;474(7350):173–8. 21614000 10.1038/nature10143 PMC3587138

[7] Chanarat S, Svasti J. Stress-induced upregulation of the ubiquitin-relative Hub1 modulates pre-mRNA splicing and facilitates cadmium tolerance in Saccharomyces cerevisiae. Biochim Biophys Acta Mol Cell Res. 2020;1867(2):118565. 31666190 10.1016/j.bbamcr.2019.118565

[8] Ammon T, Mishra SK, Kowalska K, Popowicz GM, Holak TA, Jentsch S. The conserved ubiquitin-like protein Hub1 plays a critical role in splicing in human cells. J Mol Cell Biol. 2014;6(4):312–23. 24872507 10.1093/jmcb/mju026 PMC4141198

[9] Kolathur KK, Mallya S, Barve S, Bojja SL, Wagle MM. Moonlighting functions of the ubiquitin-like protein, Hub1/UBL-5. Int J Biochem Cell Biol. 2023;162:106445. 37453225 10.1016/j.biocel.2023.106445

[10] Karaduman R, Chanarat S, Pfander B, Jentsch S. Error-Prone splicing controlled by the ubiquitin relative Hub1. Mol Cell. 2017;67(3):423–e324. 28712727 10.1016/j.molcel.2017.06.021

[11] Hossain MA, Johnson TL. Using yeast genetics to study splicing mechanisms. Methods Mol Biol. 2014;1126:285–98. 24549672 10.1007/978-1-62703-980-2_21 PMC4102252

[12] Mohammadi S, Saberidokht B, Subramaniam S, Grama A. Scope and limitations of yeast as a model organism for studying human tissue-specific pathways. BMC Syst Biol. 2015;9:96. 26714768 10.1186/s12918-015-0253-0 PMC4696342

[13] Love MI, Huber W, Anders S. Moderated Estimation of fold change and dispersion for RNA-seq data with DESeq2. Genome Biol. 2014;15(12):550. 25516281 10.1186/s13059-014-0550-8 PMC4302049

[14] Shen S, Park JW, Lu ZX, Lin L, Henry MD, Wu YN, et al. rMATS: robust and flexible detection of differential alternative splicing from replicate RNA-Seq data. Proc Natl Acad Sci U S A. 2014;111(51):E5593–601. 25480548 10.1073/pnas.1419161111 PMC4280593

[15] Langfelder P, Horvath S. WGCNA: an R package for weighted correlation network analysis. BMC Bioinformatics. 2008;9:559. 19114008 10.1186/1471-2105-9-559 PMC2631488

[16] Gasch AP, Spellman PT, Kao CM, Carmel-Harel O, Eisen MB, Storz G, et al. Genomic expression programs in the response of yeast cells to environmental changes. Mol Biol Cell. 2000;11(12):4241–57. 11102521 10.1091/mbc.11.12.4241 PMC15070

[17] DeRisi JL, Iyer VR, Brown PO. Exploring the metabolic and genetic control of gene expression on a genomic scale. Science. 1997;278(5338):680–6. 9381177 10.1126/science.278.5338.680

[18] Parenteau J, Durand M, Morin G, Gagnon J, Lucier J-F, Wellinger Raymund J, et al. Introns within ribosomal protein genes regulate the production and function of yeast ribosomes. Cell. 2011;147(2):320–31. 22000012 10.1016/j.cell.2011.08.044

[19] Szklarczyk D, Gable AL, Lyon D, Junge A, Wyder S, Huerta-Cepas J, et al. STRING v11: protein-protein association networks with increased coverage, supporting functional discovery in genome-wide experimental datasets. Nucleic Acids Res. 2019;47(D1):D607–13. 30476243 10.1093/nar/gky1131 PMC6323986

[20] Chen J, Yuan B. Detecting functional modules in the yeast protein-protein interaction network. Bioinformatics. 2006;22(18):2283–90. 16837529 10.1093/bioinformatics/btl370

[21] Edgar R, Domrachev M, Lash AE. Gene expression omnibus: NCBI gene expression and hybridization array data repository. Nucleic Acids Res. 2002;30(1):207–10. 11752295 10.1093/nar/30.1.207 PMC99122

[22] Wingett SW, Andrews S. FastQ screen: A tool for multi-genome mapping and quality control. F1000Res. 2018;7:1338. 30254741 10.12688/f1000research.15931.1 PMC6124377

[23] Kim D, Langmead B, Salzberg SL. HISAT: a fast spliced aligner with low memory requirements. Nat Methods. 2015;12(4):357–60. 25751142 10.1038/nmeth.3317 PMC4655817

[24] Li H, Handsaker B, Wysoker A, Fennell T, Ruan J, Homer N, et al. Seq Alignment/Map Format SAMtools Bioinformatics. 2009;25(16):2078–9. 10.1093/bioinformatics/btp352 PMC2723002 19505943

[25] Liao Y, Smyth GK, Shi W. FeatureCounts: an efficient general purpose program for assigning sequence reads to genomic features. Bioinformatics. 2014;30(7):923–30. 24227677 10.1093/bioinformatics/btt656

[26] Patro R, Duggal G, Love MI, Irizarry RA, Kingsford C. Salmon provides fast and bias-aware quantification of transcript expression. Nat Methods. 2017;14(4):417–9. 28263959 10.1038/nmeth.4197 PMC5600148

[27] Soneson C, Love MI, Robinson MD. Differential analyses for RNA-seq: transcript-level estimates improve gene-level inferences. F1000Res. 2015;4:1521. 26925227 10.12688/f1000research.7563.1 PMC4712774

[28] Benjamini Y, Drai D, Elmer G, Kafkafi N, Golani I. Controlling the false discovery rate in behavior genetics research. Behav Brain Res. 2001;125(1–2):279–84. 11682119 10.1016/s0166-4328(01)00297-2

[29] Anders S, Huber W. Differential expression analysis for sequence count data. Genome Biol. 2010;11(10):R106. 20979621 10.1186/gb-2010-11-10-r106 PMC3218662

[30] Katz Y, Wang ET, Silterra J, Schwartz S, Wong B, Thorvaldsdóttir H, et al. Quantitative visualization of alternative exon expression from RNA-seq data. Bioinformatics. 2015;31(14):2400–2. 25617416 10.1093/bioinformatics/btv034 PMC4542614

[31] Anders S, Reyes A, Huber W. Detecting differential usage of exons from RNA-seq data. Genome Res. 2012;22(10):2008–17. 22722343 10.1101/gr.133744.111 PMC3460195

[32] Yeo G, Burge CB. Maximum entropy modeling of short sequence motifs with applications to RNA splicing signals. J Comput Biol. 2004;11(2–3):377–94. 15285897 10.1089/1066527041410418

[33] Quinlan AR, Hall IM. BEDTools: a flexible suite of utilities for comparing genomic features. Bioinformatics. 2010;26(6):841–2. 20110278 10.1093/bioinformatics/btq033 PMC2832824

[34] Ruxton GD. The unequal variance t-test is an underused alternative to student’s t-test and the Mann–Whitney U test. Behav Ecol. 2006;17(4):688–90.

[35] Wu T, Hu E, Xu S, Chen M, Guo P, Dai Z, et al. ClusterProfiler 4.0: A universal enrichment tool for interpreting omics data. Innov (Camb). 2021;2(3):100141. 10.1016/j.xinn.2021.100141 PMC8454663 34557778

[36] Subramanian A, Tamayo P, Mootha VK, Mukherjee S, Ebert BL, Gillette MA, et al. Gene set enrichment analysis: a knowledge-based approach for interpreting genome-wide expression profiles. Proc Natl Acad Sci U S A. 2005;102(43):15545–50. 16199517 10.1073/pnas.0506580102 PMC1239896

[37] Reimand J, Isserlin R, Voisin V, Kucera M, Tannus-Lopes C, Rostamianfar A, et al. Pathway enrichment analysis and visualization of omics data using g:profiler, GSEA, cytoscape and enrichmentmap. Nat Protoc. 2019;14(2):482–517. 30664679 10.1038/s41596-018-0103-9 PMC6607905

[38] Cappadocia L, Lima CD. Ubiquitin-like protein conjugation: structures, chemistry, and mechanism. Chem Rev. 2018;118(3):889–918. 28234446 10.1021/acs.chemrev.6b00737 PMC5815371

[39] Ma S, Ming Y, Wu J, Cui G. Cellular metabolism regulates the differentiation and function of T-cell subsets. Cell Mol Immunol. 2024;21(5):419–35. 38565887 10.1038/s41423-024-01148-8 PMC11061161

[40] Koodathingal P, Staley JP. Splicing fidelity: DEAD/H-box ATPases as molecular clocks. RNA Biol. 2013;10(7):1073–9. 23770752 10.4161/rna.25245 PMC3849154

[41] Wilkinson CR, Penney M, McGurk G, Wallace M, Gordon C. The 26S proteasome of the fission yeast Schizosaccharomyces Pombe. Philos Trans R Soc Lond B Biol Sci. 1999;354(1389):1523–32. 10582238 10.1098/rstb.1999.0496 PMC1692671

[42] Parenteau J, Durand M, Véronneau S, Lacombe AA, Morin G, Guérin V, et al. Deletion of many yeast introns reveals a minority of genes that require splicing for function. Mol Biol Cell. 2008;19(5):1932–41. 18287520 10.1091/mbc.E07-12-1254 PMC2366882

[43] Tanabe K, Takei K. Dynamic instability of microtubules requires dynamin 2 and is impaired in a Charcot-Marie-Tooth mutant. J Cell Biol. 2009;185(6):939–48. 19528294 10.1083/jcb.200803153 PMC2711604

[44] Smaczynska-de R, Allwood II, Aghamohammadzadeh EG, Hettema S, Goldberg EH, Ayscough MW. A role for the dynamin-like protein Vps1 during endocytosis in yeast. J Cell Sci. 2010;123(Pt 20):3496–506. 20841380 10.1242/jcs.070508 PMC2951468

[45] Varikkapulakkal A, Ghosh A, Mishra SK. Broader roles of the ubiquitin-like protein Hub1 indicated by its yeast two-hybrid interactors. MicroPubl Biol. 2022;2022:000519. 10.17912/micropub.biology.000519. 10.17912/micropub.biology.000519 PMC8790634 35098049

[46] Borneman AR, Leigh-Bell JA, Yu H, Bertone P, Gerstein M, Snyder M. Target hub proteins serve as master regulators of development in yeast. Genes Dev. 2006;20(4):435–48. 16449570 10.1101/gad.1389306 PMC1369046

[47] Jo H, Kim T, Chun Y, Jung I, Lee D. A compendium of chromatin contact maps reflecting regulation by chromatin remodelers in budding yeast. Nat Commun. 2021;12(1):6380. 34737268 10.1038/s41467-021-26629-6 PMC8569116

[48] Hang J, Wan R, Yan C, Shi Y. Structural basis of pre-mRNA splicing. Science. 2015;349(6253):1191–8. 26292705 10.1126/science.aac8159

[49] Vethantham V, Yang Y, Bowman C, Asp P, Lee JH, Skalnik DG, et al. Dynamic loss of H2B ubiquitylation without corresponding changes in H3K4 trimethylation during myogenic differentiation. Mol Cell Biol. 2012;32(6):1044–55. 22252316 10.1128/MCB.06026-11 PMC3295016

[50] Pakos-Zebrucka K, Koryga I, Mnich K, Ljujic M, Samali A, Gorman AM. The integrated stress response. EMBO Rep. 2016;17(10):1374–95. 27629041 10.15252/embr.201642195 PMC5048378

[51] Son J, Shen SS, Margueron R, Reinberg D. Nucleosome-binding activities within JARID2 and EZH1 regulate the function of PRC2 on chromatin. Genes Dev. 2013;27(24):2663–77. 24352422 10.1101/gad.225888.113 PMC3877756

[52] Byron SA, Van Keuren-Jensen KR, Engelthaler DM, Carpten JD, Craig DW. Translating RNA sequencing into clinical diagnostics: opportunities and challenges. Nat Rev Genet. 2016;17(5):257–71. 26996076 10.1038/nrg.2016.10 PMC7097555

[53] Conesa A, Madrigal P, Tarazona S, Gomez-Cabrero D, Cervera A, McPherson A, et al. A survey of best practices for RNA-seq data analysis. Genome Biol. 2016;17:13. 26813401 10.1186/s13059-016-0881-8 PMC4728800

[54] Pervouchine DD, Knowles DG, Guigó R. Intron-centric Estimation of alternative splicing from RNA-seq data. Bioinformatics. 2013;29(2):273–4. 23172860 10.1093/bioinformatics/bts678 PMC3546801

[55] López-Maury L, Marguerat S, Bähler J. Tuning gene expression to changing environments: from rapid responses to evolutionary adaptation. Nat Rev Genet. 2008;9(8):583–93. 18591982 10.1038/nrg2398

[56] Park E, Pan Z, Zhang Z, Lin L, Xing Y. The expanding landscape of alternative splicing variation in human populations. Am J Hum Genet. 2018;102(1):11–26. 29304370 10.1016/j.ajhg.2017.11.002 PMC5777382

