# Supplementary material for: Integrative analysis of Hub1 overexpression: driving transcriptional reprogramming and alternative splicing in Saccharomyces cerevisiae
Source: BMC Genomics. 2025 Oct 6;26:885. doi: 10.1186/s12864-025-12006-w (PMC12502333; doi:10.1186/s12864-025-12006-w)
Supplement: Supplementary file 1 — Additional file 1: Supplementary Figures and Tables. Contains supplementary figures S1-S2 and supplementary table S1 with detailed legends and methodological descriptions. [file 12864_2025_12006_MOESM1_ESM.docx]

**Supplementary Materials**

Integrative Analysis of Hub1 Overexpression: Driving Transcriptional Reprogramming and Alternative Splicing in *Saccharomyces cerevisiae*

**Supplementary Figures**

**Supplementary Figure S1. Principal Component Analysis of Hub1 overexpression samples**


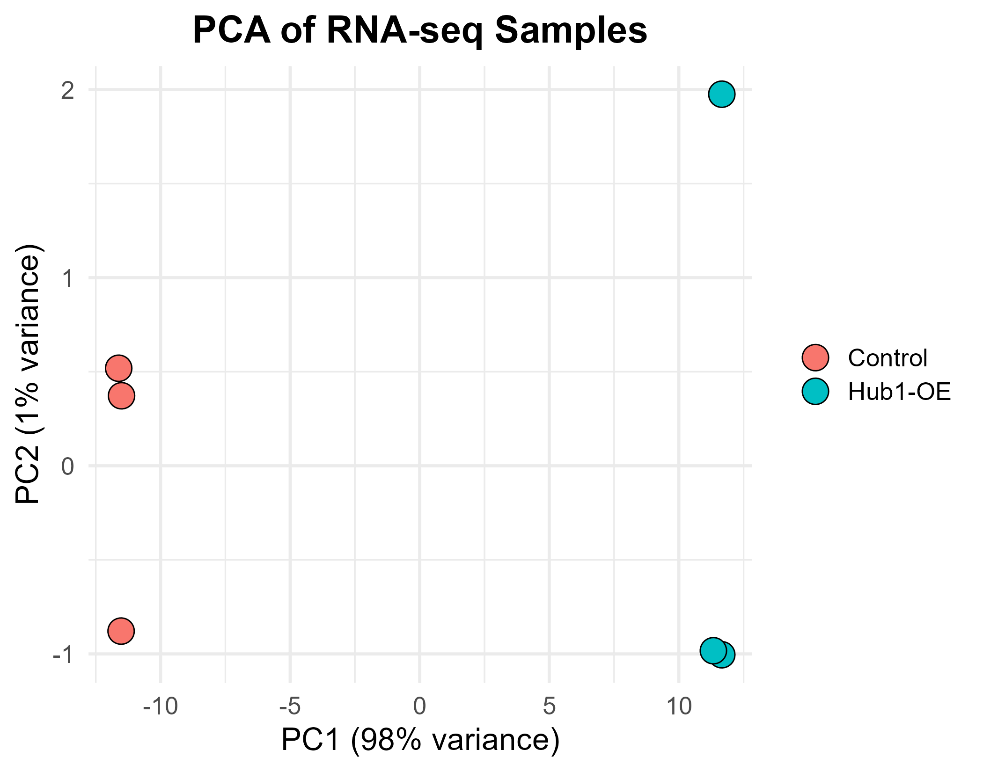


**Supplementary Fig. S1.** PCA plot of control vs. Hub1-OE RNA-seq samples. Principal component analysis was performed on variance-stabilized transformed (VST) gene expression data. The first principal component (PC1) explains 98% of the total variance and clearly separates Hub1-overexpressing samples from controls, while PC2 accounts for 1% of the variance. Biological replicates within each group cluster tightly, demonstrating high experimental reproducibility and minimal technical variability.

**Supplementary Figure S2. Differential Exon Usage Analysis**

**
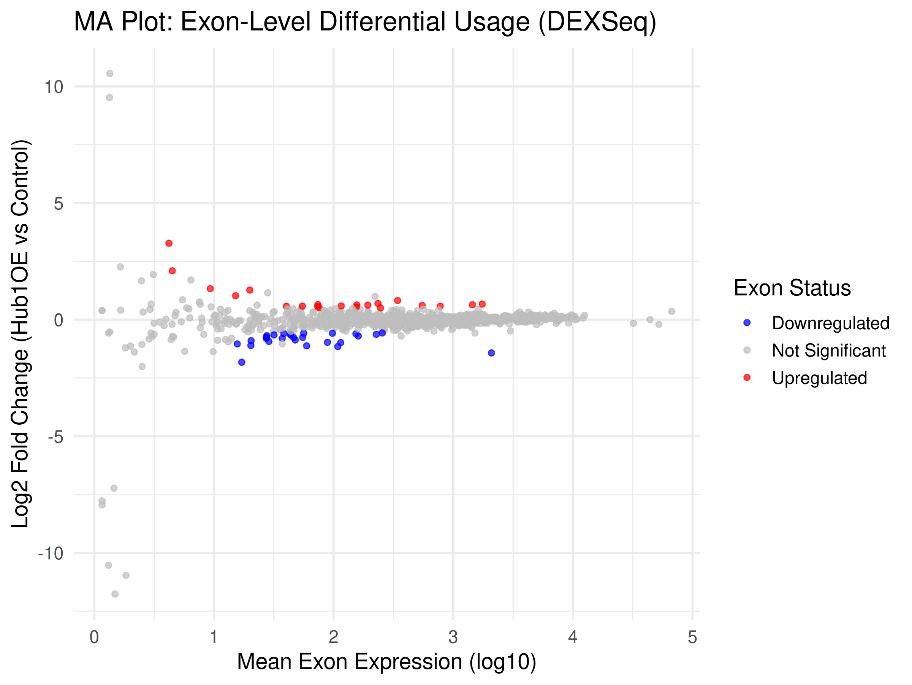
A**

**
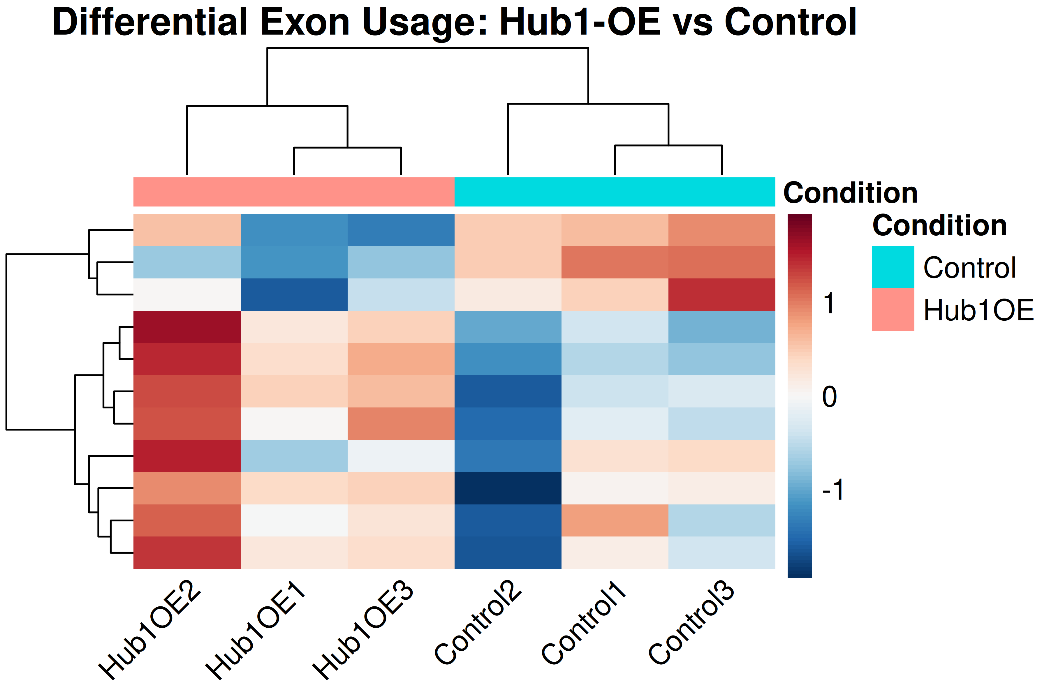
B**

**Supplementary Fig. S2. Differential Exon Usage Analysis in Hub1-OE vs. Control Yeast Cells** **(A)** MA plot of differential exon usage generated using DEXSeq. Each dot represents an exon, plotted by log2 fold change (Hub1-OE vs. Control) on the y-axis and base mean expression on the x-axis (log10 scale). Red: significantly upregulated (padj < 0.05); blue: downregulated; gray: non-significant. A total of 27 exons were significantly downregulated and 21 exons upregulated. **(B)** Heatmap of top differentially used exons (padj < 0.05). Rows represent individual exons and columns represent biological replicates. Values are log2-transformed normalized exon counts, scaled per row. Color indicates relative expression (red = high; blue = low). Dendrogram shows clustering by condition.

**Supplementary Tables**

**Supplementary Table S1. Splice Site Motif Scores for Hub1-Regulated Exons**

| **Exon ID** | **Splice Site** | **Sequence** | **MaxEnt Score** |
| --- | --- | --- | --- |
| **DYN2_exon** | 5′ donor | GGTGTAAGT | **−18.32** |
| **DYN2_exon** | 3′ acceptor | TTGTGTGTAAGTCACCAATTTTC | **−5.52** |
| YDL246C_E1 | 5′ donor | TGAATGAAA | −17.90 |
| YDL243C_E1 | 5′ donor | CGATTAATC | −10.06 |
| YDR387C_E1 | 5′ donor | CCATTGATG | −14.73 |
| YDL094C_E1 | 5′ donor | TTTTTAATT | −10.80 |
| YDR438W_E1 | 5′ donor | GAATGAATC | −15.91 |
| YDL246C_E1 | 3′ acceptor | AATATGTCTCAAAATAGTAACCC | −9.07 |
| YDL243C_E1 | 3′ acceptor | TTTATGGGCTCTATGAATAAGGA | −3.17 |
| YDR387C_E1 | 3′ acceptor | GGTATGTCTACAGATGAAAGTGA | −12.01 |
| YDL094C_E1 | 3′ acceptor | ACGATGGCAGTAAATATCAGCAA | −6.42 |
| YDR438W_E1 | 3′ acceptor | GACCTATCTTGGATGCCTAACGT | −6.73 |

**Supplementary Table S1.** Splice site motif sequences and MaxEntScan scores for Hub1-regulated exons. Splice site sequences (9 bp for 5′ donor, 23 bp for 3′ acceptor) were extracted from the *Saccharomyces cerevisiae* genome and evaluated using MaxEntScan scoring models. Lower scores indicate weaker splice sites. The DYN2 exon shows significantly weak 5′ donor site (score = −18.32, p = 0.03 compared to canonical sites) and moderately weak 3′ acceptor site (score = −5.52), consistent with Hub1's role in facilitating recognition of non-consensus splice sites.
